# Change in healthcare costs in Europe 2018–2023 (data report)

**DOI:** 10.3389/fpubh.2026.1774180

**Published:** 2026-03-09

**Authors:** Joanna Wozniak-Holecka, Julian Smółka, Karolina Sobczyk, Tomasz Holecki

**Affiliations:** 1 Department of Health Promotion, Faculty of Public Health, Medical University of Silesia in Katowice, Katowice, Poland; 2 Department of Health Economics and Management, Faculty of Public Health, Medical University of Silesia in Katowice, Katowice, Poland

**Keywords:** COVID-19 pandemic, European Union, GDP, health expenditure, health financing, OECD, out-of-pocket payments, public health economics

## 1 Introduction

The dynamics of healthcare expenditure are shaped by a complex interplay of demographic, social, and economic factors (1), as well as the specific organizational and funding structures of national health systems (2–4). While health spending in European Union (EU) nations has historically outpaced general economic growth, this trajectory has been subject to significant volatility, most notably during the financial crisis of 2008/09 (5–7) and, more recently, the global COVID-19 pandemic (8). The extent to which national economies were impacted by the SARS-CoV-2 crisis necessitated a substantial recalibration of health financing metrics, creating a divergence between health expenditure growth and economic production.

The onset of the pandemic in early 2020 severely disrupted health systems across Europe (9). Although reductions in elective surgeries and hospitalizations initially lowered spending on non-COVID-19 care (10, 11), the immediate necessity to expand treatment, testing, and diagnostic capacities introduced sharp upward pressure on total expenditure (12). This occurred simultaneously with a contraction in Gross Domestic Product (GDP) across almost every EU nation due to lockdowns and economic restrictions (13). Consequently, the average share of health spending in GDP rose significantly, increasing by 1 percentage point to 9.2% in 2020 across the EU.

Beyond the immediate crisis, European health systems currently face compounding uncertainties. These include the long-term economic challenges associated with the war in Ukraine (14), high inflation (15), and supply chain disruptions (16), all occurring against the backdrop of a progressively aging population that continues to drive demand for care (17–19).

This study provides a comparative assessment of healthcare spending trajectories in Europe over a six-year period (2018–2023). The timeframe was selected to facilitate an analysis of three distinct phases: the pre-pandemic baseline, the pandemic crisis (2020–2021), and the post-pandemic recovery period. Drawing upon data from the Organization for Economic Co-operation and Development (OECD) and adhering to the System of Health Account (SHA 2011 revised) framework to ensure international comparability, this research employs descriptive and inferential statistical analysis to address three specific research questions:

- Do healthcare spending trajectories differ statistically significantly between established Western systems, Southern mixed systems, and the “catching-up” economies of Central and Eastern Europe (CEE) during the crisis?
- To what extent did the growth in health expenditure in CEE nations exhibit a structural “catch-up” dynamic independent of annual GDP fluctuations compared to mature Western economies?
- Did a higher reliance on public financing (government schemes) provide greater stability and mitigate the volatility of health spending during the pandemic shock?

By stratifying countries into macro-regions and applying regression and correlation models, this study aims to isolate the structural determinants of health spending elasticity during the COVID-19 crisis.

## 2 Materials and methods

### 2.1 Timeframe

The study covers a six-year period from 2018 to 2023, selected to facilitate a comparative assessment of healthcare spending across three distinct phases: the pre-pandemic baseline (2018-2019), the pandemic (2020-2021) and the post-pandemic period (2022-2023). This temporal scope is designed to capture the fluctuations in health expenditure caused by the COVID-19 crisis and to identify broader, long-term trends in health financing.

### 2.2 Data types

Data on healthcare expenditure levels:

- this includes total health expenditure measured in Euro Purchasing Power Parity (PPP) per capita to allow for cross-country comparisons of spending power (Table 1);
- it also encompasses the growth rates of per capita health spending to identify trends over the analyzed period (Table 2).

**Table 1:** Health expenditure in Europe, 2023 [current prices].

**Country**	**Total health expenditure**		
	**Euros PPP (millions)**	**Euros PPP (per capita)**	**%GDP**
Austria	44,751	4,901	11.2
Belgium	53,832	4,570	10.8
Bulgaria	13,438	2,085	7.9
Croatia	7,837	2,032	7.1
Cyprus	2,677^P^	2,795^P^	8.1^P^
Czechia	31,780	2,925	8.4
Denmark	24,819	4,173	9.5
Estonia	2,939	2,145	7.5
Finland	22,310	3,995	10.5
France	298,094	4,360	11.5
Germany	450,892	5,414	11.7
Greece	22,806	2,191	8.4
Hungary	18,462	1,925	6.4
Iceland	1,506	3,905	8.7
Ireland	23,763	4,474	6.6
Italy	182,038	3,086	8.4
Latvia	3,713	1,977	7.3
Lithuania	6,935	2,415	7.3
Luxembourg	3,044	4,567	5.7
Malta	1,969	3,563	8.8
Netherlands	86,670	4,848	9.8
Norway	31,306 ^E^	5,672^E^	9.4^E^
Poland	82,192	2,240	7.1
Portugal	31,742^P^	3,001^P^	10.0^P^
Romania	33,846	1,776	5.7
Slovak Republic	11,332	2,088	7.4
Slovenia	6,618	3,121	9.3
Spain	151,680	3,137	9.2
Sweden	49,400	4,688	11.3
Switzerland	52,636	5,922	11.7
United Kingdom	278,678	4,082	11.0
EU members (average)	61,836	3,277	8.6
Non-UE members (average)	91,032	4,895	10.2

^P^Provisional value; ^E^Estimated value; ^B^Time series break; ^D^Definition differs; n/d - no data.

Source: OECD Data Explorer. Health expenditure and financing [data-explorer.oecd.org; access 01.12.2025]. Own calculations.

**Table 2:** Growth in total healthcare spending per capita in Europe 2018–2023 [current prices].

**Country**	**2018**	**2019**	**2020**	**2021**	**2022**	**2023**	**Δ%2018–23**	**Δ%2019–20**
Austria	3,891	4,004	4,095	4,789	4,834	4,901	26.0%	2.3%
Belgium	3,773	3,869	3,966	4,205	4,336	4,570	21.1%	2.5%
Bulgaria	1,290	1,334	1,556	1,823	1,871	2,085	61.6%	16.6%
Croatia	1,346	1,430	1,506	1,866	1,868	2,032	51.0%	5.3%
Cyprus	1,795	1,947	2,270	2,594	2,711^P^	2,795^P^	55.7%	16.6%
Czechia	2,179	2,324	2,666	2,863	2,759	2,925	34.2%	14.7%
Denmark	3,702	3,765	3,998	4,435	4,181	4,173	12.7%	6.2%
Estonia	1,649	1,740	1,901	2,038	1,989	2,145	30.1%	9.2%
Finland	3,021	3,102	3,231	3,461	3,619	3,995	32.3%	4.2%
France	3,575	3,678	3,821	4,103	4,207	4,360	21.9%	3.9%
Germany	4,358	4,516	4,790	5,198	5,432	5,414	24.2%	6.1%
Greece	1,615	1,652	1,726	1,879	1,994	2,191	35.7%	4.5%
Hungary	1,480	1,495	1,701	1,892	1,892	1,925	30.0%	13.8%
Iceland	2,976	3,085	3,259	3,564	3,739	3,905	31.2%	5.6%
Ireland	3,401	3,482	3,655	3,991	4,236	4,474	31.6%	5.0%
Italy	2,451	2,528	2,620	2,828	2,978	3,086	25.9%	3.6%
Latvia	1,298	1,436^B^	1,563	2,242	2,033	1,977	52.3%	8.8%
Lithuania	1,661	1,899	2,011	2,310	2,316	2,415	45.4%	5.9%
Luxembourg	3,691	3,741	3,811	4,127	4,303	4,567	23.7%	1.9%
Malta	2,635	2,958	3,026	3,322	3,363	3,563	35.2%	2.3%
Netherlands	3,824	3,933	4,238	4,694^B^	4,719	4,848	26.8%	7.8%
Norway	4,531	4,598	4,697	5,062	5,357	5,672^E^	25.2%	2.2%
Poland	1,469	1,592	1,633	1,765	1,967	2,240	52.5%	2.6%
Portugal	2,175	2,269	2,312	2,699^B^	2,884	3,001^P^	37.9%	1.9%
Romania	1,157	1,311	1,443	1,646	1,613	1,776	53.4%	10.0%
Slovak Republic	1,401	1,519	1,593	1,875	1,963	2,088	49.0%	4.8%
Slovenia	2,124	2,287	2,443	2,697	2,956	3,121	46.9%	6.8%
Spain	2,428	2,544	2,599	2,826	2,978	3,137	29.2%	2.2%
Sweden	3,803	3,855	3,947	4,245	4,380	4,688	23.3%	2.4%
Switzerland	4,770	4,897	4,957	5,384	5,706	5,922	24.1%	1.2%
United Kingdom	2,940	3,042	3,500	3,711	3,885	4,082	38.8%	15.1%
EU members (average)	2,489	2,600	2,745	3,052	3,125	3,277	31.7%	5.6%
Non-UE members (average)	3,804	3,905	4,103	4,430	4,672	4,895	28.7%	5.1%

^P^Provisional value; ^E^Estimated value; ^B^Time series break; ^D^Definition differs, n/d - no data.

Source: OECD Data Explorer. Health expenditure and financing [data-explorer.oecd.org; access 01.12.2025]. Own calculations.

Data on healthcare financing relative to the economy:

- information regarding health expenditure expressed as a percentage of Gross Domestic Product (GDP) (Table 3).

**Table 3:** Growth in health expenditure as a share of GDP in Europe 2018–2023.

**Country**	**2018**	**2019**	**2020**	**2021**	**2022**	**2023**	**Δ%2018–23**	**Δ%2019–20**
Austria	10.5	10.6	11.4	12.2	11.2	11.2	6.1%	7.4%
Belgium	11.0	10.9	11.5	11.3	10.7	10.8	−1.7%	5.6%
Bulgaria	7.3	7.1	8.4	8.6	7.6	7.9	7.9%	18.6%
Croatia	6.7	6.8	7.7	8.1	7.3	7.1	6.4%	13.2%
Cyprus	6.8	7.1	8.8	9.5	9.0 ^P^	8.1 ^P^	18.9%	25.1%
Czechia	7.4	7.5	9.0	9.2	8.5	8.4	14.3%	19.8%
Denmark	10.1	10.2	10.7	10.7	9.5	9.5	−6.2%	5.0%
Estonia	6.6	6.6	7.5	7.5	6.9	7.5	13.8%	12.4%
Finland	9.1	9.2	9.7	9.9	9.7	10.5	15.1%	5.2%
France	11.3	11.2	12.1	12.2	11.8	11.5	1.7%	8.6%
Germany	11.2	11.4	12.5	12.7	12.4	11.7	5.0%	9.1%
Greece	8.1	8.1	9.4	9.0	8.4	8.4	4.0%	15.6%
Hungary	6.6	6.2	7.2	7.3	6.6	6.4	−2.8%	15.8%
Iceland	8.4	8.6	9.6	9.7	9.1	8.7	3.2%	11.7%
Ireland	6.7	6.6	7.0	6.4	6.0	6.6	−2.5%	5.3%
Italy	8.7	8.6	9.6	9.3	8.9	8.4	−2.9%	11.0%
Latvia	6.4	6.9	7.5	9.4	8.1	7.3	13.6%	9.4%
Lithuania	6.5	6.9	7.4	7.7	7.2	7.3	13.1%	6.8%
Luxembourg	5.3	5.5	5.8	5.6	5.6	5.7	7.5%	5.5%
Malta	8.6	9.1	10.7	10.6	9.8	8.8	3.4%	17.4%
Netherlands	9.9	9.9	11.0	11.1 ^B^	10.0	9.8	−0.2%	10.2%
Norway	10.0	10.4	11.4	9.8	7.9	9.4 ^E^	n/d	9.4%
Poland	6.3	6.4	6.4	6.4	6.5	7.1	14.2%	0.6%
Portugal	9.4	9.5	10.5	11.2 ^B^	10.5	10.0^P^	6.4%	10.6%
Romania	5.5	5.7	6.2	6.5	5.8	5.7	3.5%	9.0%
Slovak Republic	6.6	6.9	7.1	7.6	7.7	7.4	10.8%	2.2%
Slovenia	8.4	8.6	9.5	9.5	9.6	9.3	11.4%	10.8%
Spain	9.1	9.2	10.8	10.3	9.7	9.2	1.7%	17.1%
Sweden	11.1	11.0	11.4	11.3	10.9	11.3	1.4%	4.4%
Switzerland	11.2	11.4	12.0	12.0	11.6	11.7	4.8%	5.2%
United Kingdom	9.8	10.0	12.1	12.1	11.1	11.0	12.2%	21.0%
EU members (average)	8.2	8.3	9.1	9.3	8.8	8.6	5.4%	10.3%
Non-UE members (average)	9.8	10.1	11.3	10.9	9.9	10.2	3.7%	11.6%

^P^Provisional value; ^E^Estimated value; ^B^Time series break; ^D^Definition differs; n/d, no data.

Source: OECD Data Explorer. Health expenditure and financing [data-explorer.oecd.org; access 01.12.2025]. Own calculations.

Data on financing schemes:

- a breakdown of funding sources, specifically distinguishing between government schemes, compulsory contributory health insurance, voluntary healthcare payment schemes, and household out-of-pocket payments (Table 4);
- as well as additional focus on household out-of-pocket payments as a percentage of total health expenditure (Table 5).

**Table 4:** Health expenditure by type of financing in Europe (%), 2023.

**Country**	**Government schemes**	**Compulsory contributory health insurance schemes**	**Voluntary health insurance schemes**	**Household out-of-pocket payments**
Austria	33.1	43.5	6.9	16.5^D^
Belgium	18.4	55.2	4.9	21.5
Bulgaria	15.4	47.6	1.5	35.5
Croatia	7.6	77.4	5.7	9.4
Cyprus	19.7 ^P^	57.1 ^P^	5.2^P^	17.9^P^
Czechia	11.8	72.7	0.9	14.6
Denmark	83.3	–	2.7	14.0
Estonia	9.0	66.9	2.1	22.1
Finland	68.7	12.3	4.9	14.1
France	4.4	80.0	6.3	9.3
Germany	7.9	78.0	3.0	11.1
Greece	28.6	32.3	4.6	34.3
Hungary	11.6	62.1	3.2	23.1
Iceland	83.6	–	1.7	14.7
Ireland	76.0	0.6	12.1	11.3
Italy	72.9	0.2	3.3	23.6
Latvia	59.6	–	5.3	35.1
Lithuania	7.1	60.2	1.6	31.0
Luxembourg	6.9	78.7	4.4	8.8
Malta	66.0	0.1	3.1	30.9
Netherlands	8.1	74.6	5.3	12.0
Norway	n/d	n/d	n/d	n/d
Poland	11.0	66.6	6.6	15.8
Portugal	58.9^P^	2.7^P^	9.2^P^	29.3^P^
Romania	14.3	61.9	0.8	23.0
Slovak Republic	4.0	75.0	0.9	20.2
Slovenia	8.8	64.8	14.0	12.4
Spain	69.5	3.7	6.0	20.9
Sweden	86.1	–	1.0	13.0
Switzerland	22.9	44.6	8.9	22.0
United Kingdom	81.2	–	4.1	14.6
EU members (average)	32.2	43.5	4.6	19.6
Non-UE members (average)	62.6	14.9	4.9	17.1

^P^Provisional value; ^E^Estimated value; ^B^Time series break; ^D^Definition differs; n/d, no data.

Source: OECD Data Explorer. Health expenditure and financing [data-explorer.oecd.org; access 01.12.2025].

**Table 5:** Household out-of-pocket payments (% of total health expenditure) in Europe 2018–2023.

**Country**	**2018**	**2019**	**2020**	**2021**	**2022**	**2023**	**Δ%2018–23**	**Δ%2019–20**
Austria	19.3	18.8	17.2	16.0	16.3	16.5^D^	−14.6%	−8.4%
Belgium	19.4	20.6	19.3	20.3	21.4	21.5	11.0%	−6.3%
Bulgaria	39.3	37.8	35.5	33.7	35.1	35.5	−9.7%	−6.0%
Croatia	11.4	11.5	10.4	9.4	9.2	9.4	−17.7%	−8.9%
Cyprus	44.4	33.7	21.4	16.2^B^	18.0^P^	17.9^P^	−59.6%	−36.6%
Czechia	14.2	14.3	11.1	12.5	14.3	14.6	3.1%	−22.6%
Denmark	13.8	13.8	13.4	13.3	13.2	14.0	1.1%	−3.0%
Estonia	24.5	23.9	21.4	22.2	23.1	22.1	−9.8%	−10.4%
Finland	18.4	17.4	16.5	16.7	16.1	14.1	−23.3%	−5.5%
France	10.4	10.3	9.4	9.2	9.2	9.3	−11.4%	−8.7%
Germany	12.2	12.4	11.3	11.4	10.4	11.1	−9.2%	−8.4%
Greece	36.1	33.6	33.4	33.3	33.5	34.3	−4.9%	−0.6%
Hungary	26.8	27.8	26.1	24.6	24.0	23.1	−13.9%	−5.9%
Iceland	15.8	15.4	14.9	14.5	13.7	14.7	−7.2%	−3.6%
Ireland	12.7	12.7	11.4	11.5	11.6	11.3	−10.7%	−9.6%
Italy	24.0	23.9	21.5	22.8	23.3	23.6	−1.3%	−10.0%
Latvia	39.2	35.1	31.9	27.0	30.7	35.1	−10.5%	−9.2%
Lithuania	31.5	32.3	28.7	29.9	31.8	31.0	−1.6%	−11.2%
Luxembourg	10.4	9.6	8.4	8.8	8.7	8.8	−15.9%	−12.5%
Malta	34.3	34.1	30.3	29.7	30.0	30.9	−9.8%	−11.2%
Netherlands	10.8	10.4	9.3	10.9 ^B^	11.9	12.0	11.5%	−11.0%
Norway	14.0	13.9	13.8	13.9	14.1	n/d	n/d	−0.9%
Poland	20.4	20.1	19.5	19.8	18.4	15.8	−22.4%	−2.8%
Portugal	29.9	30.6	28.0	29.1 ^B^	29.1	29.3 ^P^	−2.0%	−8.5%
Romania	19.5	18.9	19.0	20.9	21.4	23.0	18.0%	0.8%
Slovak Republic	18.9	19.2	18.8	19.4	19.3	20.2	6.6%	−2.1%
Slovenia	12.0	11.7	12.5	12.9	12.4	12.4	3.4%	7.0%
Spain	24.1	23.8	21.7	21.3	21.0	20.9	−13.6%	−9.2%
Sweden	14.2	13.9	12.9	13.1	13.1	13.0	−8.9%	−7.2%
Switzerland	21.8	21.8	20.7	21.4	21.7	22.0	0.8%	−5.1%
United Kingdom	16.2	16.0	12.4	13.0	14.4	14.6	−9.9%	−22.5%
EU members (average)	21.9	21.2	19.3	19.1	19.5	19.6	−10.4%	−9.1%
Non-UE members (average)	17.0	16.8	15.4	15.7	16.0	17.1	0.9%	−8.0%

^P^Provisional value; ^E^Estimated value; ^B^Time series break; ^D^Definition differs; n/d, no data.

Source: OECD Data Explorer. Health expenditure and financing [data-explorer.oecd.org; access 01.12.2025]. Own calculations.

### 2.3 Data collection

The primary data were retrieved from the Organization for Economic Co-operation and Development (OECD) databases, specifically using the OECD Data Explorer under the “Health expenditure and financing” category. To ensure international comparability and consistency across different national health systems, the data collection methodology aligns with the System of Health Account (SHA 2011 revised) framework (20).

The SHA 2011 revised framework is a global statistical standard developed in collaboration by the OECD, Eurostat, and the World Health Organization to ensure high international comparability of health spending data. The framework employs a tri-axial system that cross-classifies health expenditures according to three core dimensions:

- classification of Health Care Functions (ICHA-HC);
- classification of Health Care Providers (ICHA-HP);
- classification of Health Care Financing Schemes (ICHA-HF) (20).

As indicated in the framework “While health systems can vary significantly among countries, SHA aims to enhance international health care expenditure data by delineating the boundary of health care according to a functional classification.” It “proposes a framework for the systematic description of the financial flows related to health care” (20).

The dataset integrates information from both OECD member countries and non-OECD European economies (such as Bulgaria, Romania, and Croatia). The analysis incorporates finalized historical data as well as provisional and estimated values for the most recent years (2022–2023) where final figures were not yet fully ratified.

As the OECD does not directly provide GDP per capita PPP data in euros and it would be necessary to imply it based on per capita health expenditure and the share of health expenditure in GDP, which would lead to unwanted simplifications, GDP per capita data for statistical analysis purposes is sourced directly from Eurostat (21). After trial calculations, the data is consistent with reasonable discrepancies.

### 2.4 Data analysis

The analysis relies primarily on numerical statistics to evaluate health financing across European nations. The data were processed to ensure comparability across different economic contexts using purchasing power parity (PPP) adjustment. The analysis includes:

- time-trend analysis – used to evaluate growth trajectories of health spending per capita and as a share of GDP over the six-year period (2018–2023) (allowing to identify specific fluctuations caused by the COVID-19 pandemic);
- comparative analysis – used to assess of disparities in spending levels between high-income Western/Northern European countries and Central/Eastern European countries, as well as differences between EU member states and non-EU economies;
- structural analysis of financing – used to investigate the composition of health funding, particularly calculating the proportional shares of government schemes, compulsory insurance, voluntary schemes, and household out-of-pocket payments;
- economics impact assessment – used to examine the relationship between country's general economic performance (GDP growth) and health expenditure, in particular during the economic slowdown associated with the pandemic.

To move beyond descriptive comparisons, inferential statistical methods were employed to test the study's hypotheses.

First, One-Way Analysis of Variance (ANOVA) was conducted for each year from 2018 to 2023 to determine if the variation in health spending (as a percentage of GDP) between the three defined macro-regions (Western/Northern Europe, Southern Europe, and CEE – Table 6) was statistically significant.

**Table 6:** Categorization of countries for comparative analysis.

**Group/Region**	**Economic & systemic characteristics**	**List of countries**
Western & Northern Europe	•High GDP per capita •Stable, mature health systems (Bismarck/Beveridge models) •Health expenditure typically >10% of GDP •Slower but stable growth	Austria, Belgium, Denmark, Finland, France, Germany, Iceland, Ireland, Luxembourg, Netherlands, Norway, Sweden, Switzerland
Central & Eastern Europe (CEE)	•Lower but rapidly growing GDP •Systems under transformation or reform •Health expenditure typically 6–8% of GDP •High growth dynamics	Bulgaria, Croatia, Czechia, Estonia, Hungary, Latvia, Lithuania, Poland, Romania, Slovakia, Slovenia
Southern Europe	•Medium/High GDP but high sensitivity to economic crises •High share of Out-of-Pocket (OOP) payments •Often underfunded relative to needs despite National Health Service models	Cyprus, Greece, Italy, Malta, Portugal, Spain

Source: own elaboration.

Second, a multivariate Ordinary Least Squares (OLS) regression model was constructed to analyze the elasticity of health spending relative to economic growth. The model took the form:


$$\begin{array}{l} \text{Growt}{\text{h}}_{\text{Health}}={\text{β}}_{0}+{\text{β}}_{1}\text{Growt}{\text{h}}_{\text{GDP}}+{\text{β}}_{2}\text{Region}\\ \;+{\text{β}}_{3}\left(\text{Growt}{\text{h}}_{\text{GDP}}\times \text{Region}\right)+\text{ϵ} \end{array}$$

This interaction model allowed us to test the “catch-up” hypothesis by isolating the baseline growth rate of health spending for each region while controlling for GDP performance.

Finally, Pearson correlation analysis was performed to assess the relationship between the depth of public financing (share of government schemes in 2019) and the stability of health expenditure, defined as the volatility (standard deviation) of spending growth during the crisis period (2018–2023).

All analyses were performed using Stata/SE 19.5, with statistical significance defined at *p* < 0.05.

## 3 Results and explanation

### 3.1 Health expenditure per capita

Numerous demographic (22), social, and economic factors (23), as well as the funding arrangements and organizational structure of the health system itself, influence a nation's health spending level and how it evolves over time (4). As demonstrated by the COVID-19 pandemic, the degree to which a nation was affected by the crisis may also have an impact on total spending levels (24).

Given these factors, there are large variations in the level and growth of health spending across Europe. In 2023 with spending at EUR 5,923 per person, Switzerland was the biggest spender in Europe, followed by Norway (EUR 5,677.8) and Germany (EUR 5,414). Spending levels in Austria, Netherlands, Sweden, Belgium, Luxembourg, France, Ireland, Denmark and Finland were also well above the EU average of EUR 3,277. At the other end of the scale, Romania, Hungary, Bulgaria, Croatia and Greece were the lowest spending countries in the EU, well below the EU average. The average for non-EU European countries was EUR 4,895 (Table 1). Analysis of the data indicates that on a per capita basis, there is a three-fold difference in health spending between high-income countries in Western and Northern Europe and some low spending countries in Central and Eastern Europe.

The analysis confirmed that the observed differences in health spending across Europe are structural rather than random. One-way ANOVA tests revealed statistically significant heterogeneity in health spending as a percentage of GDP across the three macro-regions for every year analyzed (*p* < 0.001). For the pre-pandemic baseline (2019), the variance between regions was highly significant [*F*_(2, 28)_ = 14.31, *p* < 0.001]. This structural divergence persisted through the peak of the pandemic in 2020 (*F* = 12.18, *p* < 0.001) and the recovery phase in 2023 (*F* = 9.81, *p* < 0.001), indicating that the pandemic did not homogenize European health spending patterns but rather exacerbated existing regional stratifications.

### 3.2 Growth in health expenditure per capita

Growth in per capita health spending reached an average of 31.7% in EU member states between 2018 and 2023. Outside the European Union, it was 28.7% (Table 2). This growth in UE ranged from very moderate growth values of 12.7% in Denmark, through 21.9% in France and 24.2% in Germany, to significant increases in per capita health spending of more than 50% in Poland, Latvia, Croatia, Romania and even up to 61.6% in Bulgaria.

Health systems were severely disrupted by the COVID-19 epidemic that began in early 2020. Because there were fewer hospitalizations and elective surgeries, the amount spent on care unrelated to COVID-19 decreased. However, the crisis forced nations to quickly implement new resources throughout the health sector, such as expanding hospital treatment capacity and testing and diagnostic capacities. Reserving treatment capacity for COVID-19 patients in certain countries resulted in significant incentives for healthcare providers. As a result, upward pressure on health spending in 2020 can be seen in all of the EU member states (Table 2). In 2020 per capita health spending increased on average by 5.6% in EU countries compared to 2019. Among EU member states, per capita health spending increased by about 14–17% in Hungary, Bulgaria, the Czech Republic and Cyprus. Switzerland (1.2%), Luxembourg (1.9%) and Portugal (1.8%) saw the lowest growth in per capita spending over the 2019–2020 period. The average growth in expenditure for non-EU countries in the period under review was 5.1%.

### 3.3 Health expenditure in relation to GDP

The proportion of a country's healthcare expenditure relative to all other goods and services in the economy, as well as its temporal variations, is influenced by both the magnitude of health spending and the overall economic size (4, 25). During the 1990s and early 2000s EU countries generally saw health spending outpace the rest of the economy (26), leading to an almost continual rise in the ratio of health expenditure to gross domestic product (GDP), but this trend was disrupted with the financial and economic crisis of 2008/09 (7, 8). The COVID-19 pandemic caused significant disparities in the growth trajectories of health expenditure and economic production, necessitating a substantial recalibration of this metric (24).

In 2023, 8.6% of the GDP of the European Union was devoted to healthcare (Table 1). Germany and France dedicated the high share to health at over 11% of their respective GDP. Austria also spent 11.2% of their GDP on health. The lowest shares of the overall economic output allocated to health were in Luxembourg (5.7%), Romania (5.7%), Hungary (6.4%) and Ireland (6.6%). In Poland, 7.1% of GDP was spent on health during the period under review. Across Europe, Switzerland was additional high spender on health (with shares at 11.7%).

Growth in the capital expenditure on health as a share of GDP reached an average of 5.4% in EU member states between 2018 and 2023. Outside the European Union it was 3.7% (Table 3). This change in expenditure in the EU ranged from visible decrease in Denmark (−6.2%), through a small one in Hungary, Ireland, Italy and Belgium ranging from −3% to −1.5%, up to moderate growth in Germany, Greece and Austria (4%−6.1%) and significant one of 12–15% in the Czech Republic, Slovenia, Estonia and Croatia. Cyprus (18.9%) and Finland (15.1%) recorded the highest increase in expenditure relative to GDP.

The COVID-19 problem had substantial repercussions for the rise of economic and health expenditures in 2020. The monitoring and treatment of COVID-19 patients, as well as the expansion of treatment capacity, brought about a new range of direct and indirect expenditures for the health system during the pandemic. At the same time, the GDP of almost every EU nation fell due to lockdowns and limitations on economic activity. Between 2019 and 2020, average health spending per capita increased by 5.6% in EU member states (Table 2), while GDP per capita fell sharply by an average of 5.5% over the same period (13). As a result, the share of health spending in GDP increased significantly by 0.8 percentage point to 9.1% in 2020 across the European Union.

Data for 2023 show further increases in health spending, which can be seen for most EU member states. After 2023, further uncertainties emerge as EU Member States face growing economic challenges due to the war in Ukraine, high inflation and supply chain disruptions. The progressive aging of the population also has significant impact on healthcare expenditure (27).

The regression analysis identified a significant “catch-up” dynamic in Central and Eastern European (CEE) economies. The model explained 9.6% of the variance in spending growth [*F*_(5, 145)_ = 3.10, *p* = 0.01]. Crucially, the regional dummy variable for Western/Northern Europe showed a significant negative coefficient (β = −4.90, *p* = 0.001) relative to the CEE baseline.

This implies that, holding GDP growth constant, mature Western health systems exhibited a baseline spending growth rate that was approximately 4.9 percentage points lower than that of CEE nations. This statistically confirms the hypothesis that CEE countries were driven by a structural necessity to modernize and expand their health systems (the “catch-up effect”), leading to high expenditure growth independent of immediate economic performance. In contrast, Southern European systems showed a smaller, marginally significant negative baseline effect (β = −3.43, *p* = 0.054).

### 3.4 Financing of health expenditure

In 2023, more than 75% of total healthcare spending in the EU was financed by governments and mandatory insurance (Table 4). In Sweden, Luxembourg, Croatia and Germany, government systems covered about 85% of all healthcare spending. In France, Germany and Luxembourg, compulsory health insurance financed more than three-quarters of all healthcare spending. The share of healthcare spending financed by households out-of-pocket was 19.6% in all EU countries. In three EU countries – Bulgaria, Greece and Latvia - household out-of-pocket payments accounted for at least a third of all health spending in 2023. In Slovenia and Ireland alone, voluntary health insurance financed more than 10% of health spending, compared to an EU average of 4.6%.

The decline in the share of household out-of-pocket payments in total health expenditures reached an average of 10.4% in EU member states between 2018 and 2023, outside the European Union it was 0.9% (Table 5). The decline in the EU ranged from moderate values of 12–19% in Austria, Finland, Germany, Ireland, Luxembourg, Spain and Croatia to a significant decline of more than 22% in Poland and Finland, and almost 60% in Cyprus. Seven countries recorded an increase in the analyzed category of healthcare spending expressed as a percentage of total spending. These were Belgium, the Czech Republic, Lithuania, Slovakia, Slovenia, Romania and the Netherlands.

Due to the COVID-19 health crisis household out-of-pocket payments generally fell in 2019–2020 as a result of postponement and reduced demand for elective healthcare services and patients' hesitation to seek care for fear of infection. The decline for all EU countries was 9.1% (Table 5). In Poland, the decline was 2.8%, while outside the European Union it was 8.0%.

The correlation analysis provided evidence for the stabilizing role of public financing. A moderate negative correlation (*r* = −0.40, *p* < 0.05) was found between the share of government financing in 2019 and the volatility (standard deviation) of health spending growth over the 2018–2023 period. Countries with high public coverage (e.g., Scandinavia, Germany) experienced smoother spending trajectories, whereas systems with a higher reliance on out-of-pocket payments (e.g., Bulgaria, Latvia) exhibited significantly higher volatility and “shock” spikes in expenditure, reflecting a lower capacity to absorb crisis-driven costs within existing fiscal frameworks (Tables 6–8).

**Table 7:** One-way ANOVA results testing differences in Health Spending (% GDP) across macro-regions (2018–2023).

**Year**	***F*-statistic**	***p*-value**	**Interpretation**
2018	14.54	< 0.001	Significant regional difference
2019	14.31	< 0.001	Significant regional difference
2020	12.18	< 0.001	Significant regional difference
2021	7.72	0.002	Significant regional difference
2022	7.37	0.003	Significant regional difference
2023	9.81	< 0.001	Significant regional difference

Source: Own calculations based on OECD & Eurostat data.

**Table 8:** Multivariate OLS regression.

**Variable**	**Coefficient β**	**Std. Error**	***t*-value**	***p*-value**
Constant (Base: CEE Region)	9.10	1.24	7.34	< 0.001
GDP Growth	−0.12	0.12	−1.03	0.306
Region: Western/Northern	−4.91	1.46	−3.36	0.001
Region: Southern	−3.43	1.76	−1.95	0.054
Interaction: GDP x West	0.20	0.14	1.44	0.152
Interaction: GDP x South	0.27	0.18	1.48	0.140

Model Fit: R^2^ = 0.096; F_(5, 145)_ = 3.10, *p* = 0.011.

Source: own calculations.

## Data Availability

The original contributions presented in the study are included in the article/supplementary material, further inquiries can be directed to the corresponding author.

## References

[1] Future trends in health care expenditure: A modelling framework for cross-country forecasts. [OECD Health Working Papers]. (2017).

[2] Bichay N. Health insurance as a state institution: The effect of single-payer insurance on expenditures in OECD countries. Soc Sci Med. (2020) 265:113454. doi: 10.1016/j.socscimed.2020.113454 33190928

[3] Rapoport J Jacobs P Jonsson E. Cost Containment and Efficiency in National Health Systems: A Global Comparison. Hoboken, NJ: John Wiley & Sons. (2008). 250 doi: 10.1002/9783527622962

[4] Meskarpour Amiri M Kazemian M Motaghed Z Abdi Z. Systematic review of factors determining health care expenditures. Health Policy Technol. (2021) 10:100498. doi: 10.1016/j.hlpt.2021.01.004

[5] Morgan D Astolfi R. Financial impact of the GFC: health care spending across the OECD. Health Econ Policy Law. (2015) 10:7–19. doi: 10.1017/S1744133114000218 25662194

[6] Martin A Lassman D Whittle L Catlin A. Recession contributes to slowest annual rate of increase in health spending in five decades. Health Aff. (2011) 30:11–22. doi: 10.1377/hlthaff.2010.1032 21209433

[7] Hartman M Martin A Nuccio O Catlin A. Health spending growth at a historic low in 2008. Health Aff. (2010) 29:147–55. doi: 10.1377/hlthaff.2009.0839 20048374

[8] Thomson S García-Ramírez JA Akkazieva B Habicht T Cylus J Evetovits T. How resilient is health financing policy in Europe to economic shocks? Evidence from the first year of the COVID-19 pandemic and the 2008 global financial crisis. Health Policy. (2022) 126:7–15. doi: 10.1016/j.healthpol.2021.11.002 34857406 PMC8591973

[9] Fatani M Shamayleh A Alshraideh H. Assessing the disruption impact on healthcare delivery. J Prim Care Community Health. (2024) 15:21501319241260351. doi: 10.1177/21501319241260351 38907592 PMC11193933

[10] Best MJ McFarland EG Anderson GF Srikumaran U. The likely economic impact of fewer elective surgical procedures on US hospitals during the COVID-19 pandemic. Surgery. (2020) 168:962–7. doi: 10.1016/j.surg.2020.07.014 32861440 PMC7388821

[11] Mazzaferro DM Patel V Asport N Stetson RL Rose D Plana N . The financial impact of COVID-19 on a surgical department: The effects of surgical shutdowns and the impact on a health system. Surgery. (2022) 172:1642–50. doi: 10.1016/j.surg.2022.08.014 36123177 PMC9388446

[12] OECD. Latest health spending trends: Navigating beyond the recent crises. (2024). Available online at: https://www.oecd.org/content/dam/oecd/en/publications/reports/2024/12/latest-health-spending-trends_7332c460/df0bb1ba-en.pdf (Accessed February 26, 2026).

[13] Eurostat. Real GDP per capita. ec.europa.eu/eurostat (Accessed December 1, 2025).

[14] Mohiuddin AK. Escalation of war and conflicts among the COVID-19 pandemic, natural disasters, and economic crises: A global health concern. Am J Biopharmacy Pharm Sci. (2023) 3:5. doi: 10.25259/AJBPS_21_2022

[15] Movsisyan A Wendel F Bethel A Coenen M Krajewska J Littlecott H . Inflation and health: a global scoping review. Lancet Glob Health. (2024) 12:e1038–48. doi: 10.1016/S2214-109X(24)00133-5 38762284

[16] National National Academies of Sciences Engineering and Medicine; Health and Medicine Division. “Causes and Consequences of Medical Product Supply Chain Failures,” In Shore C, Brown L, Hopp WJ, editors. Building Resilience into the Nation's Medical Product Supply Chains. Washington, DC: National Academies Press (2022). 36070409

[17] Wypych-Slusarska A Krupa-Kotara K Słowinski J Yanakieva A Grajek M. The impact of polycrisis on healthcare systems—analyzing challenges and the role of social epidemiology. Healthcare. (2025) 13:1998. doi: 10.3390/healthcare13161998 40868618 PMC12385510

[18] Ogugua JA Muonde M Maduka CP Olorunsogo TO Omotayo O. Demographic shifts and healthcare: A review of aging populations and systemic challenges. Int J Sci Res Arch. (2024) 11:383–95. doi: 10.30574/ijsra.2024.11.1.0067

[19] Khan HTA Addo KM Findlay H. Public health challenges and responses to the growing ageing populations. Public Health Chall. (2024) 3:e213. doi: 10.1002/puh2.213 40496520 PMC12039680

[20] OECD Eurostat World Health Organization. A System of Health Accounts 2011: Revised edition. OECD (2017).

[21] Eurostat. Gross domestic product (GDP) and main components per capita (2025). Available online at: https://ec.europa.eu/eurostat/databrowser/product/page/namq_10_pc (Accessed February 26, 2026).

[22] de Meijer C Wouterse B Polder J Koopmanschap M. The effect of population aging on health expenditure growth: a critical review. Eur J Ageing. (2013) 10:353–61. doi: 10.1007/s10433-013-0280-x 28804308 PMC5549212

[23] Vargas Bustamante A Shimoga SV. Comparing the income elasticity of health spending in middle-income and high-income countries: the role of financial protection. Int J Health Policy Manag. (2017) 7:255–63. doi: 10.15171/ijhpm.2017.83 29524954 PMC5890070

[24] OECD European Union. Health at a Glance: Europe 2022: State of Health in the EU Cycle. OECD Publishing (2022).

[25] Rana RH Alam K Gow J. Health expenditure and gross domestic product: causality analysis by income level. Int J Health Econ Manag. (2020) 20:55–77. doi: 10.1007/s10754-019-09270-1 31313127

[26] Kanavos P Yfantopoulos J. “Cost containment and health expenditure in the EU: a macroeconomic perspective.” *Health Care and Cost Containment in the European Union*. New York: Routledge (1999).

[27] OECD. OECD Data Explorer. Available online at: https://data-explorer.oecd.org/ (Accessed December 1, 2025).

